# Earliest Known Use of Marine Resources by Neanderthals

**DOI:** 10.1371/journal.pone.0024026

**Published:** 2011-09-14

**Authors:** Miguel Cortés-Sánchez, Arturo Morales-Muñiz, María D. Simón-Vallejo, María C. Lozano-Francisco, José L. Vera-Peláez, Clive Finlayson, Joaquín Rodríguez-Vidal, Antonio Delgado-Huertas, Francisco J. Jiménez-Espejo, Francisca Martínez-Ruiz, M. Aranzazu Martínez-Aguirre, Arturo J. Pascual-Granged, M. Mercè Bergadà-Zapata, Juan F. Gibaja-Bao, José A. Riquelme-Cantal, J. Antonio López-Sáez, Marta Rodrigo-Gámiz, Saburo Sakai, Saiko Sugisaki, Geraldine Finlayson, Darren A. Fa, Nuno F. Bicho

**Affiliations:** 1 Departamento de Prehistoria y Arqueología, Facultad de Geografía e Historia, Universidad de Sevilla, Sevilla, Spain; 2 Laboratorio de Arqueozoología, Departamento de Biología, Universidad Autónoma de Madrid, Madrid, Spain; 3 Fundación Cueva de Nerja, Nerja, Malaga, Spain; 4 Museo Municipal Paleontológico de Estepona, Estepona, Málaga, Spain; 5 The Gibraltar Museum, Gibraltar, United Kingdom; 6 Department of Social Sciences, University of Toronto, Toronto, Canada; 7 Departamento de Geodinámica y Paleontología, Facultad de Ciencias Experimentales, Huelva, Spain; 8 Instituto Andaluz de Ciencias de la Tierra Consejo Superior de Investigaciones Científicas, Universidad de Granada, Armilla, Granada, Spain; 9 Departamento de Física Aplicada I, Escuela Técnica Superior de Ingeniería Agronómica, Universidad de Sevilla, Sevilla, Spain; 10 Seminari d'Estudis i Recerques Prehistòriques, Departamento de Prehistoria, Historia Antigua y Arqueología, Facultad de Geografía e Historia, Universidad de Barcelona, Barcelona, Spain; 11 Departamento de Arqueología del Spanish Scientific Research Council, Barcelona, Spain; 12 Grupo de Investigación Arqueobiología, Instituto de Historia, Centro de Ciencias Humanas y Sociales, Spanish Scientific Research Council, Madrid, Spain; 13 Institute of Biogeosciences, Japan Agency for Marine-Earth Science and Technology, Yokosuka, Kanagawa, Japan; 14 Faculdade de Ciências Humanas e Sociais, Universidade do Algarve, Faro, Portugal; Institut de Biologia Evolutiva - Universitat Pompeu Fabra, Spain

## Abstract

Numerous studies along the northern Mediterranean borderland have documented the use of shellfish by Neanderthals but none of these finds are prior to Marine Isotopic Stage 3 (MIS 3). In this paper we present evidence that gathering and consumption of mollusks can now be traced back to the lowest level of the archaeological sequence at Bajondillo Cave (Málaga, Spain), dated during the MIS 6. The paper describes the taxonomical and taphonomical features of the mollusk assemblages from this level Bj_19_ and briefly touches upon those retrieved in levels Bj_18_ (MIS 5) and Bj_17_ (MIS 4), evidencing a continuity of the shellfishing activity that reaches to MIS 3. This evidence is substantiated on 29 datings through radiocarbon, thermoluminescence and U series methods. Obtained dates and paleoenvironmental records from the cave include isotopic, pollen, lithostratigraphic and sedimentological analyses and they are fully coherent with paleoclimate conditions expected for the different stages. We conclude that described use of shellfish resources by Neanderthals (*H. neanderthalensis*) in Southern Spain started ∼150 ka and were almost contemporaneous to Pinnacle Point (South Africa), when shellfishing is first documented in archaic modern humans.

## Introduction

Recent claims that intensive shellfish collecting is a trait of anatomically modern humans appear to be in conflict with previous studies on Neanderthal sites [1]. Indeed documentation of the occurrence of coastal, even marine, animals in Neanderthal sites dates back to the analyses of Garrod *et al.* in 1928, at Devil's Tower, Gibraltar [2]. Most of this evidence, however, has been gathered over the last decade on the western European rim of the Mediterranean Sea and the Atlantic coast of the Iberian Peninsula, with a concentration of sites on the southern fringe of the latter (Table 1). Also, with the exception of one controversial site [1], none of these reports proceed beyond MIS 3, being all post 50 ka. Up until now it has been repeatedly entertained, yet impossible to prove, that such absence of pre-50 ka data on shellfishing may have been a consequence of the restricted visibility [3] of coastal resources, due to the submergence of the Pleistocene coastlines that took place during the Holocene Transgression. Alternatively, such dearth may reflect an “economic” constraint whereby the low amplitude of the tides in most of the Mediterranean shores dictates a most restrictive development of the productive littoral zone, where most of the shellfish resources concentrate [4]. In the context of these scenarios, the data from Bajondillo Cave, presented here, provide evidence for the exploitation of coastal resources by Neanderthals at a much earlier time than any of those previously reported.

**Table 1:** Overview of Neanderthal sites with reported mollusk remains.

Country	Region	Site	References
Greece	Peloponese	Lakonis	[62]
Italy	Liguria	Costa dei Balzi Rossi	[63]
		Riparo Mocchi	
		Barma Grande	
	Latium	Grotta dei Moscerini	
	Puglia	Grotta dell'Alto	
		Grotta del Cavallo	
		Grotta del U'zzo	
		Grotta Mario Bernadini	
		Grotta dei Gigante	
Spain	Murcia	Cueva Perneras	[64]–[65]
		Hoyo de los Pescadores	
		Cueva de los Aviones	
		Cueva Antón	
	Andalusia	Abrigo 3 del Humo	[66]
		Abrigo 4 del Humo	
		Cueva Bajondillo	This paper
Gibraltar		Devil's Tower	[1]
		Gorham's Cave	[67]
		Vanguard Cave	[68]
Portugal	Algarve	Ibn Ammar	[69]
		Vale Boi	[70]
	Estremadura	Figueira Brava	[71]
		Furninha	[71]

### The archaeological site of Bajondillo Cave

**Figure 1:**
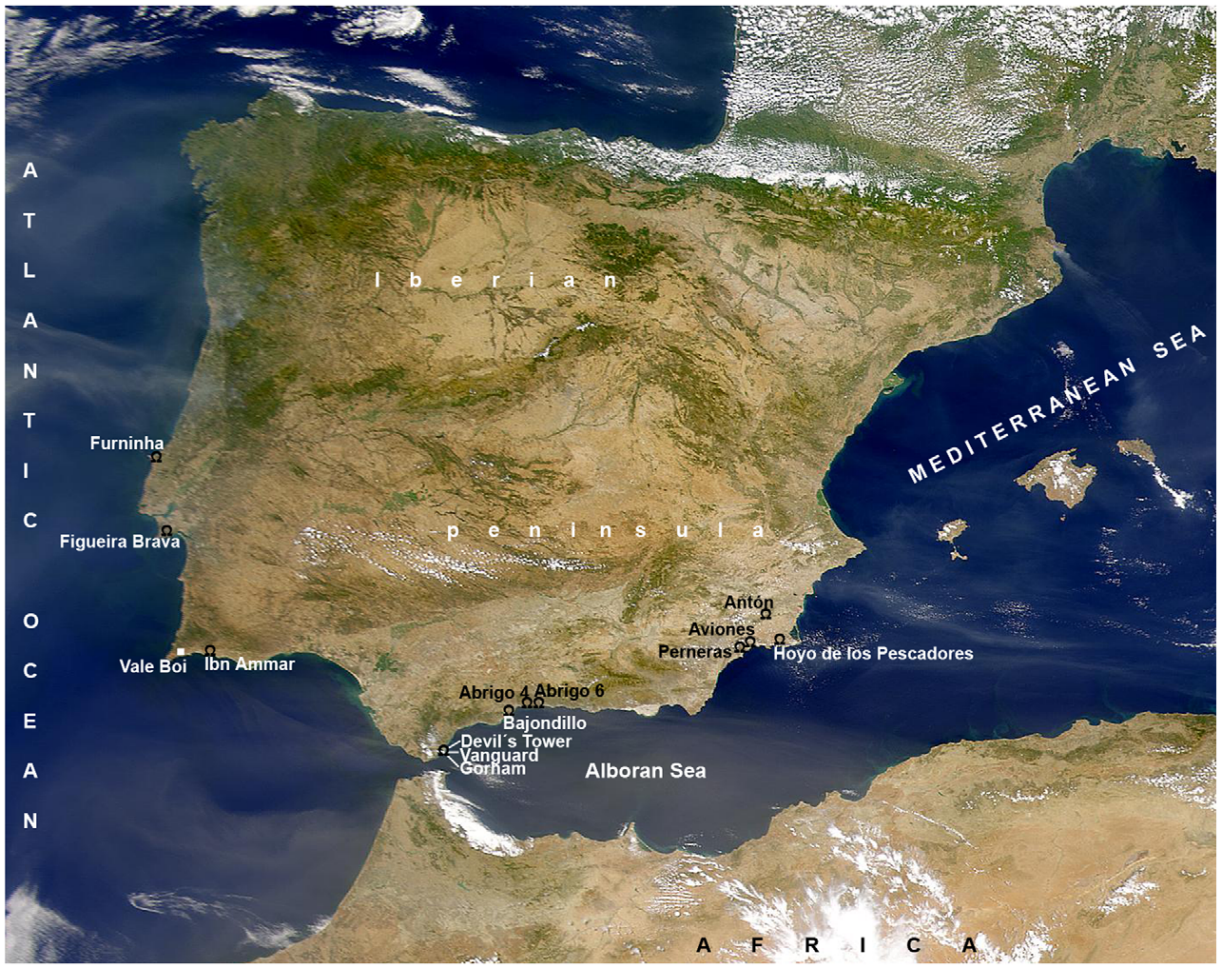
Map of the Iberian Peninsula locating Middle Paleolithic sites with reported finds of marine mollusks.

Bajondillo Cave is a *ca.* 30 m long rock shelter that opens within a 30 m high travertine formation in the city of Torremolinos (Málaga, Spain). Located some 250 m from the present-day coastline, in the centre of a sector of the southern Iberian littoral dotted with Neanderthal sites (Figure 1), the cave escaped all high sea stands by virtue of its elevation (+15 m above mean sea level, a.s.l.). Indeed, during MIS 5, the largest of the marine transgressive episodes in this sector of the Iberian coastline have been documented at a maximum of +2 m a.s.l., and the transgressive episode previous to the onset of the archaeological sequence at Bajondillo Cave (i.e., MIS 7), reached to a maximum of +4.5 m a.s.l. [5]. For these reasons, none of the stratigraphic horizons at Bajondillo Cave could have ever had a marine origin.

**Figure 2:**
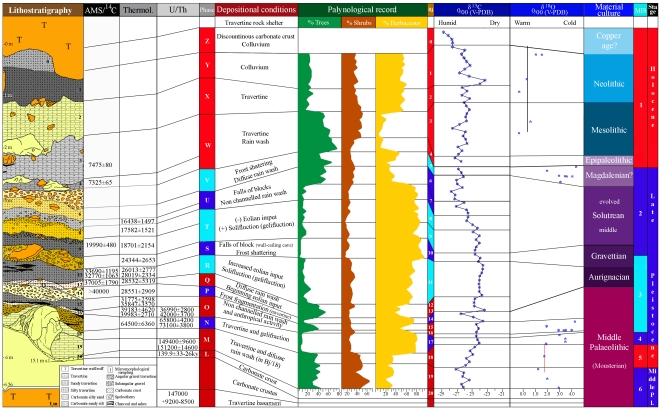
Bajondillo Cave: Overview of records from the chronostratigraphical sequence (techno-typological sequence taken from [**6**]).

The sedimentary sequence at Bajondillo Cave is a 5.4 m high deposit filling a cavity within which 20 archaeological levels (Figure 2) with significant anthropological input, that includes an abundance of lithic industries, bones, shells and hearths have been recognized. In 1989, when the cave was discovered, the stratigraphic sequence uncovered the seventeen uppermost levels (Bj_1_–Bj_17_) after excavation of 5.6 m^3^ of sediment. In 2000 and 2002, 0.06 m^3^ of sediment were additionally retrieved during sampling works that uncovered the lowermost three levels (Bj_18_–Bj_20_). Twenty nine absolute dates obtained through ^14^C/AMS, thermoluminescence (TL) and U/Th methods set the chronology of the archaeological sequence [6] and allow one to define a succession of chrono-cultural episodes that range from the Middle Paleolithic (MIS 6) to the Neolithic (MIS 1) (Figure 2; Table 2).

**Table 2:** Radiocarbon (^14^C/AMS), Thermoluminescence (TL) and Uranium/Thorium (U/Th) dates from Bajondillo Cave.

Level	U/Th (8 dates)	TL (13 dates)	AMS (8 dates)	Material	Laboratory
Bj_0_–Bj_2_	-	-	-	-	-
Bj_3_	-	-	7475±80	Charcoal	Ua-18269
Bj_4_	-	-	7325±65	Charcoal	Ua-21999
Bj_5_	-	-	-	-	-
Bj_6_	-	-	-	-	-
Bj_7_	-	16438±1497	-	Carbonate	MAD-3927
Bj_8_	-	17582±1521	-	Carbonate	MAF-3926
Bj_9_	-	18701±2154	-	Flint	MAD-2405
	-	-	19990±480	Flint	AA34710
Bj_10_	-	24344±2653	-	Flint	MAD-2470
Bj_11_	-	26013±2777	-	Flint	MAD-2482
	-	28019±2334	-	Flint	MAD-2559
	-	-	33690±1195	Sediment	Ua-17150
	-	-	32770±1065	Sediment	Ua-18050
Bj_13_	-	28532±5319	-	Carbonate	MAD-2377
	-	-	37005±1790	-	Ua-18270
Bj_14_	-	-	>40000	-	Ua-16859
	-	28551±2909	-	Flint	MAD-2463
Bj_15_	-	-	29165±725	Charcoal	Ua-18051
	-	35847±3570	-	Flint	MAD-2446
	-	31775±2598	-	Carbonate	MAD-2410
		39183±4.620		Carb. Flint	MAD-2383
Bj_16_	-	39983±2710	-	Carb. Flint	MAD-2392
	42000±3700	-	-	Bone	MA0506-1
	36900±2800	-	-	Bone	MA0506-2
Bj_17_	-	64500±6360	-	Flint	MAD-2473
	65800±4200	-		Bone	MA0505-2
	73100±3800	-	-	Bone	MA0505-1
Bj_18_	-	-	-	-	-
Bj_19_	149400±9600	-	-	Bone	MA0507-2
	151200±14600	-	-	Bone	MA0507-1
Bj_20_	139900±33000–26000	-	-	Stalag.Crust	CERAK-6483
Travertine	147000±9200–8500	-	-	Travertine	CERAK-6484

Given the tectonic stability of the sector of the Iberian coastline where Bajondillo Cave is located, one can reliably estimate its distance to the Mediterranean Sea during the various MIS stages [5], [7]. In particular, it is relevant to remark that during Bj_19_ such distance was similar to today's (ie., 200 m), indicating that humans did not have to travel far to reach the shore.

## Results and Discussion

### Archaeological data

**Figure 3:**
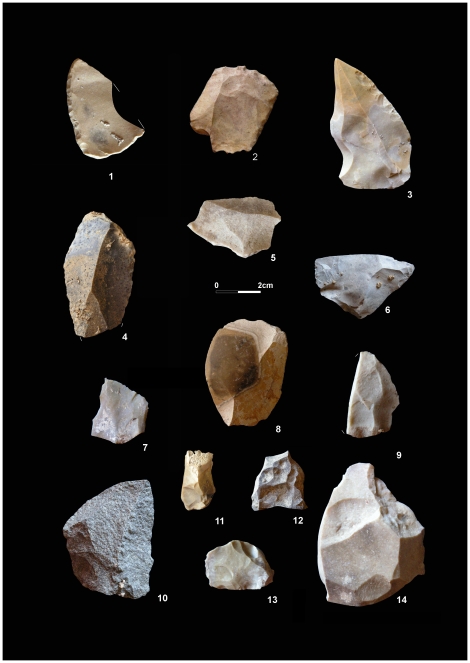
Lithic industries from Bj_19_. Retouched tools (1–4, 6, 10), Flakes (5, 7–9, 12), Levallois core (13), Thermoaltered items (6,12,14). All pieces executed in flint except no. 10 (quartzite).

The lithic industries from Bj_19_ (n = 73) are mostly flint dominated. The techno-typological characteristics of this collection (Figure 3) place it fully within the Southern Iberian Middle Paleolithic complex [6]. Flaking technology includes Levallois, discoidal and Kombewa systems, and among the retouched artifacts, one chopper and four flaked retouched tools were found. From a functional standpoint, both thermo-alterations, a recurrent feature on many of the animal remains, and sedimentary conditions have intensively altered the surface of the artifacts. Temperature-induced changes (ie., cracking, polish, etc.) preclude a detailed use wear analysis of the lithic industries. Still, unequivocal use wear has been recorded on at least three items from Bj_19_. The microspatial analysis revealed a direct association existing between the dispersion of the lithic assemblages and the faunal remains, mostly on the bottom of Bj_19_ and its contact with Bj_20_.

### Archaeozoological data

**Figure 4:**
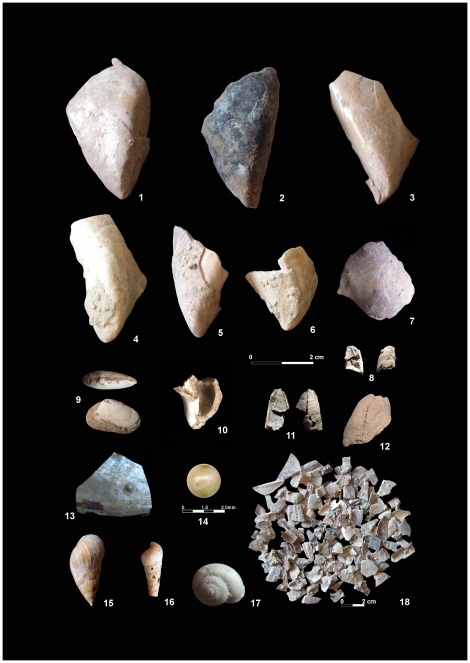
Marine mollusks and barnacles from Bj_19_. *Mytilus galloprovincialis* (1–6), *Glycymeris* sp (7), *Balanus trigonus* (8,11–12), *Donacilla cornea* (9), *Stramonita haemastoma* (10), Pearls of *M. galloprovincialis* (13–14), *Melanopsis laevigata* (15), *Rumina decollate* (16), *Iberus marmoratus* (17), Fragments of *M. galloprovincialis* (18).

Associated with the lithic industries of the Bj_19_ deposit, intensively fragmented, occasionally butchered and often burned remains of four mammal species (aurochs, red deer, wild goat and rabbit), along with nine categories of marine invertebrates, have been documented (Table 3): five of the invertebrates were identified to species level (the barnacle *Balanus trigonus*, the snail *Stramonita haemastoma* and the bivalves *Mytilus galloprovincialis, Donacilla cornea* and *Panopea glycymeris*). Two bivalves (*Glycymeris* sp. and *Thracia* sp.) and one barnacle (*Balanus* sp.) were identified to genus level, and two mollusk remains to Class level (i.e., marine snails, Gastropoda). The mussel *M. galloprovincialis* is the overwhelmingly-dominant species at Bj_19_, both in terms of identified remains and individuals, although the extensive fracturing that these shells exhibit (Figure 4:1–6) dictates that 1,247 remains should more properly remain identified to family level (i.e., Mytilidae). On the basis of current habitats [8]–[9], the vast majority of the marine mollusks would have been collected from significantly to moderately exposed rocky shores and from sandy beaches, easily achieved during daily low tides.

**Table 3:** Continental and marine (bold) shellfish species from Bajondillo Cave levels _19-17_.

Taxon	NR	NISP	LI
*Melanopsis laevigata* (Lamarck, 1822)	4	4	
*Iberus marmoratus* (Férussac, 1821)	1	1	
*Rumina decollata* (Linné, 1758)	1	1	
*Theba pisana* (Müller, 1774)	2	2	
*Xerotricha* sp.	1	1	
Helicidae sp.	2	2	
***Mytilus galloprovincial*** **. (Lamarck, 1819)**	94	3	
***Acanthocardia tuberculata*** ** (Linné, 1758)**	1	1	
***Callista chione*** ** (Linné, 1758)**	1	1	
TOTAL Bajondillo 17	107	16	319
*Melanopsis laevigata* (Lamarck, 1822)	11	11	
*Rumina decollata* (Linné, 1758)	10	10	
*Succinea putris* (Linné, 1758)	1	1	
*Vitrea* sp.	1	1	
*Otala lactea* (Müller, 1774)	2	2	
*Cecilioides acicula* (Müller, 1774)	3	3	
*Theba pisana* (Müller, 1774)	3	3	
*Bitynia tentaculata* (Linné, 1758)	15	15	
*Helicidae* sp.	6	6	
***Mytilus galloprovincial.*** ** (Lamarck, 1819)**	496	10	
***Balanus*** ** sp.**	4	4	
TOTAL Bajondillo 18	552	66	91
*Melanopsis laevigata* (Lamarck, 1822)	11	11	
*Iberus marmoratus* (Férussac, 1821)	1	1	
*Succinea putris* (Linné, 1758)	2	2	
*Cecilioides acicula* (Müller, 1774)	1	1	
*Theba pisana* (Müller, 1774)	3	3	
Helicidae sp.	7	7	
*Rumina decollata* (Linné, 1758)	10	10	
*Otala lactea* (Müller, 1774)	1	1	
***Stramonita haemastoma*** ** (Linné, 1758)**	1	1	
***Balanus trigonus*** ** (Dawin, 1854)**	2	2	
***Balanus*** ** sp.**	3	3	
Gastropoda	2	2	
***Donacilla cornea*** ** (Poli, 1795)**	1	1	
***Glycymeris*** ** sp.**	5	3	
***Mytilus galloprovincial*** **. (Lamarck 1819)**	1305	29	
***Thracia*** **? sp.**	1	1	
***Panopea glycimeris*** ** (von Born, 1778)**	1	1	
*Pisium casertatum* (Poli, 1791)	1	1	
**Pearl**	1	-	
TOTAL Bajondillo 19	1359	80	73

NR: Number of rest. NISP: Number of individual species. LI: number of lithic industries.

Of particular interest are the contrasting taphonomical signatures of the marine mollusks when compared to their corresponding terrestrial equivalents. In this way, and despite their fragility and generally small size, the shells of the pulmonates appear in good condition and complete for the most part (i.e., 59% at Bj_19_; 79% at Bj_18_ and 64% at Bj_17_), bearing no traces of alterations suggestive of human manipulation (Figure 4: 15–17). In contrast, almost all of the marine mollusks exhibit intensive mechanical fracturing, with sharp edges on their shells suggestive of an absence of post-depositional transport, and very few appear complete (i.e., barely 7% at Bj_19_). Such fracturing, coupled with the absence of shells eroded by water, indicates that the marine mollusks from Bajondillo Cave, and in particular those from Bj_19_ do not represent “background fauna” from the nearby beach, a phenomenon that has recurrently caused problems in the association of early Middle Paleolithic shellfish deposits from the Mediterranean with paleo-human activities. In addition, a substantial percentage of the mussels exhibit burning marks (Figure 4: 1–6). These are recorded on 48% of the adult specimens from Bj_19_, the young mussels never exhibiting such traces. Thermo-alterations suggest consumption rather than passive burning, given that in most cases only the outer portions of the shells appear carbonized and/or flaked. An indirect line of evidence supporting this same hypothesis is provided by five of the epibiont barnacle remains that fire not only detached from the mussel shells but that in that process were thoroughly carbonized, as is the case of the four specimens from Bj_18_ (Figure 4: 8,11) or else calcined, as happens with the specimen from Bj_19_ (Figure 4: 12).

Unequivocal evidence for the transport of complete specimens to the cave in this earliest moment of the occupation is provided by a complete specimen of the fragile *Donacilla cornea* (Figure 4: 9). Fine sieving also provided indirect evidence of a transport of fresh animals in the form of a pearl whose morphometrics identify as deriving from a mussel (Figure 4:14); the excellent state of preservation of this fragile specimen reinforces the notion of a lack of long distance transport. Mussel shells with pearls in formation have been also occasionally retrieved from this same level (Figure 4: 13).

Marine mollusk gathering at Bajondillo Cave appears by no means restricted to this early moment of the Mousterian that level Bj_19_ represents. The presence of 590 marine shell fragments at Bj_18_ and Bj_17_, representing a minimum number of 19 individuals, hints at the prevalence of a practice that reached up to MIS 4, and where mussels kept on being the main cropped taxon (Table 3).

Continuity of the shellfishing practice has been also documented at the level of processing. Fracturing appears to have followed a similar pattern throughout the Middle Paleolithic, aiming at the removal of the densest portions of the shells as is the case of the apex in gastropods (Figure 4: 10) and the umbo in bivalves (Figure 4: 1–7). Consistency is also revealed in the burning marks. In this way, the 55% of the adult mussels from Bj_18_ that were burned exhibit the same pattern recorded on those from Bj_19_, and, again, none of the young specimens from Bj_18_ exhibit thermo-alterations. The low number of burned mussels from level Bj_17_ does not lend them to comparison but may simply reflect the scarcity of marine shells therein retrieved (Tables 3, 4). Indeed, one of the prominent features of the marine mollusk assemblages at Bajondillo Cave is their drastic decline throughout the Middle Paleolithic. Such decline, reaching to MIS 3 -a stage not considered in our paper due to its abundant documentation of Neanderthal shell collecting activities in the northern Mediterranean (paper in preparation) contrasts with the increase in the number of lithic implements (Table 4), and as these incorporate tens of thousands of items throughout the sequence, we are positive that the drop in shellfish remains does not reflect a less intensive occupation of the cave. The single one factor that best correlates with this decline is the increasing distance of the cave to the coast. That distance reached to *ca.* 2.5 km in Bj_18_ (MIS 5) and went up to *ca.* 8.0 km in Bj_17_ (MIS 4) [6]. Studies carried out on shellfish transport by *H. sapiens* evidence that foragers rarely carry shell over more than 5–10 km [10]–[11]. One can thus postulate that at Bj_17_ Bajondillo Cave was reaching that distance threshold from the shore above which remains of seashells were more likely to be left on the beach than be transported back to the site.

**Table 4:** Estimated densities of shellfish and lithic industries from Bajondillo Cave.

	m_2_	m_3_	NR	NISP	NR Marine Shells/m^2^	NISP Marine Shells/m^3^	Lithic industries remains	Lithic industries/m^2^	Lithic industries/m^3^
Bj_17_	0.62	0.031							
Bj_18_	0.15	0.0075							
Bj_19_	0.24	0.017							
Bj_17_ Continental shellls			11	11	17.7	354.8			
Bj_17_ Marine shells			96	5	154.8	161.3			
**Bj_17_ Total**			**107**	**16**	**172.6**	**516.1**	**319**	**514.5**	**10290.3**
Bj_18_ Continental shells			52	52	346.7	6933.3			
Bj_18_ Marine shells			500	14	3333.3	1866.7			
**Bj_18_ Total**			**552**	**66**	**3680.0**	**1866.7**	**91**	**606.7**	**12133.3**
Bj_19_ Continental shells			37	37	154.2	2176.5			
Bj_19_ Marine shells			1322	41	5508.3	2411.8			
**Bj_19_ Total**			**1359**	**78**	**5662.5**	**4587.3**	**73**	**304.2**	**4291.1**

Excavated areas (m^2^) and volumes (m^3^). NR: Number of rest. NISP: Number of individual species.

Combined [11]–[12], the heavy incidence of burning, the selection of particular species common in shell middens (as opposed to beach deposits), the selective burning and fracture patterns, the presence of intact fragile shells, together with the association with lithics and butchered mammal remains, indicate that humans, rather than animals, were the agents responsible for the mollusk accumulations at Bajondillo Cave.

### Chronology

The Bajondillo Cave chronological sequence is now safely secured through 29 datings carried out by three different methods (i.e., ^14^C/AMS, TL and U/Th series, Table 2); Six U/Th datings from levels Bj_19_, Bj_17_ and Bj_16_ were made on bones, with two subsamples analyzed per bone (Table 5). Table 5 provides activity concentrations in both mBq/g and activity ratios. In its last three columns, the dates for each bone sample according to each of the three uptake models are given. It should be remarked that U/Th datings are coincident with TL datings carried out on a flint artefact from level Bj_17_ (MAD2473) and on another flint artefact plus its carbonate encrusting from Bj_16_ [i.e., MAD2383 (carbonate) and MAD2392 (flint)] (Figure 2, Table 2).

**Table 5:** U/Th dates from levels Bj_19_, Bj_17_ and Bj_16_ at Bajondillo Cave.

Layer	Sample	^238^U	^234^U	^230^Th	^234^U/^238^U	^230^Th/^234^U	EU (ka)	LU84 (ka)	LU4 (ka)
**Bj_16_**	MA0506-1	272±8	302±9	51.2±2.0	1.111±0.017	0.1696±0.0085	20.1±1.1	**42.0±3.7**	21.6±1.3
	MA0506-2	258±8	287±9	45.2±1.2	1.115±0.019	0.1575±0.0067	18.6±0.9	**36.9±2.8**	19.8±1.1
	TL = 40.0±2.7	-	-	-	-	-	-	-	-
**Bj_17_**	MA0505-1	234±7	266±8	73.0±1.8	1.135±0.017	0.2745±0.0106	34.6±1.6	**73.1±3.8**	37.7±2.1
	MA0505-2	243±8	273±9	69.3±1.6	1.123±0.019	0.2543±0.0104	31.7±1.5	**65.8±4.2**	34.2±2.0
	TL = 61.5±6.4	-	-	-	-	-	-	**-**	-
**Bj_19_**	MA0507-1	269±8	322±9	149.9±7.8	1.197±0.018	0.4650±0.0276	66.5±5.4	**151.2±14.6**	76.1±7.2
	MA0507-2	273±8	324±10	148.8±4.0	1.186±0.018	0.4600±0.0189	65.6±3.6	**149.4±9.6**	74.5±5.1

The data on Table 5 evidence that U and Th concentrations in the two subsamples from each bone are quite similar considering the error bars (1σ) and provide nominal ages concordant with each other. In addition, ^234^U/^238^U activity ratios in the bone samples are similar, though slightly higher, to those found in the carbonate samples from the same area.

As can be seen, EU ages are much younger than the expected ages, even when one considers that two of them are in the range of expected concordance (≤50 ka). As expected, LU4 ages are younger than LU84 ages, and the later are concordant with TL ages as well as with an U/Th age taken at the bottom of the sequence (i.e., 147 ka). These data evidence that the bones have been continuously absorbing U isotopes. The similarity in the ^234^U/^238^U activity ratios between the bones and carbonates of the Bajondillo Cave area demonstrates that, by comparison with ^238^U, ^234^U was not preferentially absorbed by bone. Indeed, what these results evidence is that the mechanism of U uptake by bones in this system was produced by a linear uptake of both U isotopes through time.

**Figure 5:**
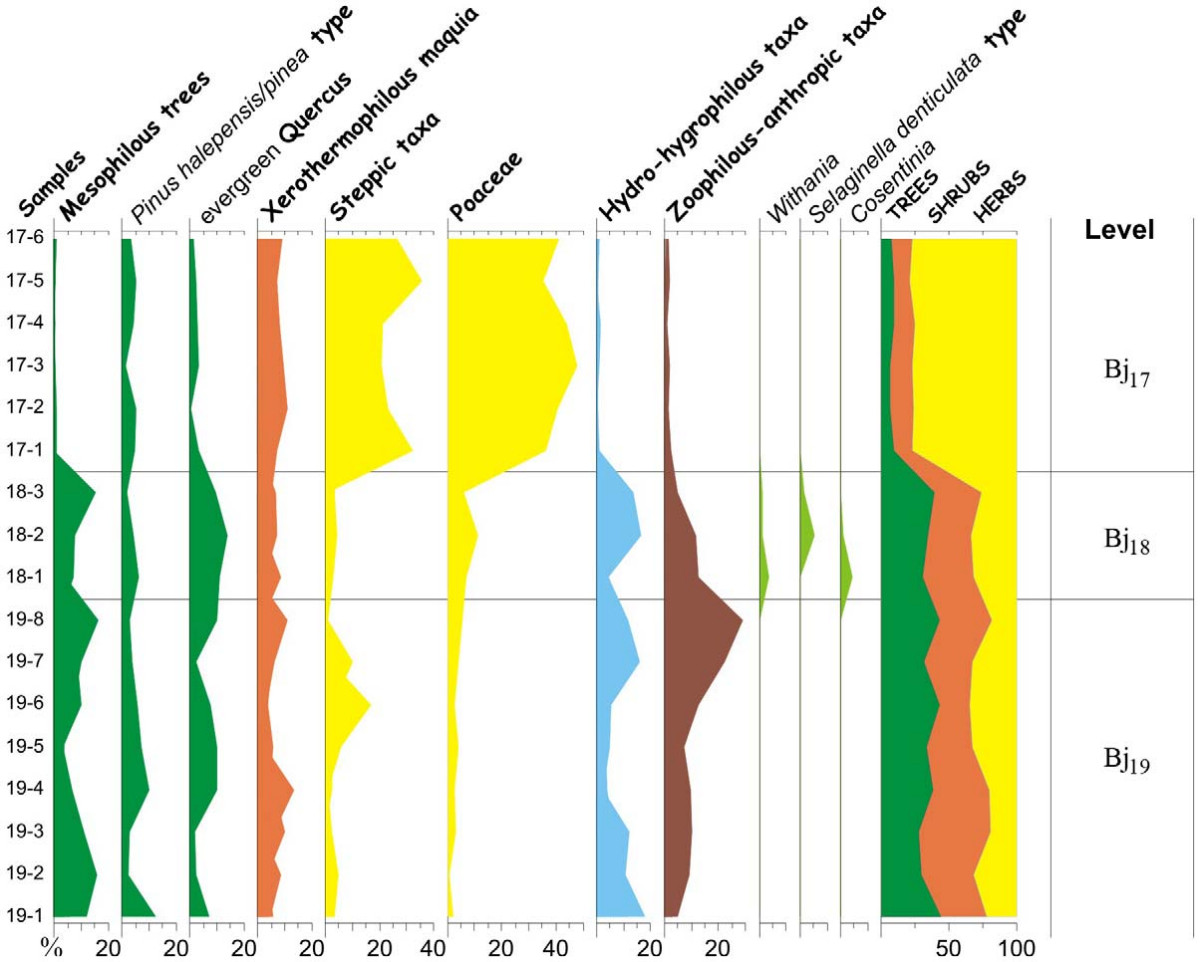
Bajondillo Cave: Pollen sequence from levels Bj_19_-Bj_17_.

The date of the cave's basal travertine sets the lower limit of this sequence (Table 5). The flowstone overlaying the basal travertine (Bj_20_-U/Th: 139.9±33–36 ka) is particularly relevant since its synchronicity is statistically identical with that of the lowermost of the archaeological levels (i.e., Bj_19_-U/Th: 150.3±10 ka; average from two datings, 1σ). The absence of absolute dates from level Bj_18_ does not preclude its assignment to MIS 5 as the depositional conditions of the sediments and the pollen indicate a moment of climatic amelioration that would have been in all cases previous to 95 ka (Figures 2, 5; see Materials and Methods).

### Isotopic and geochemical records

**Figure 6:**
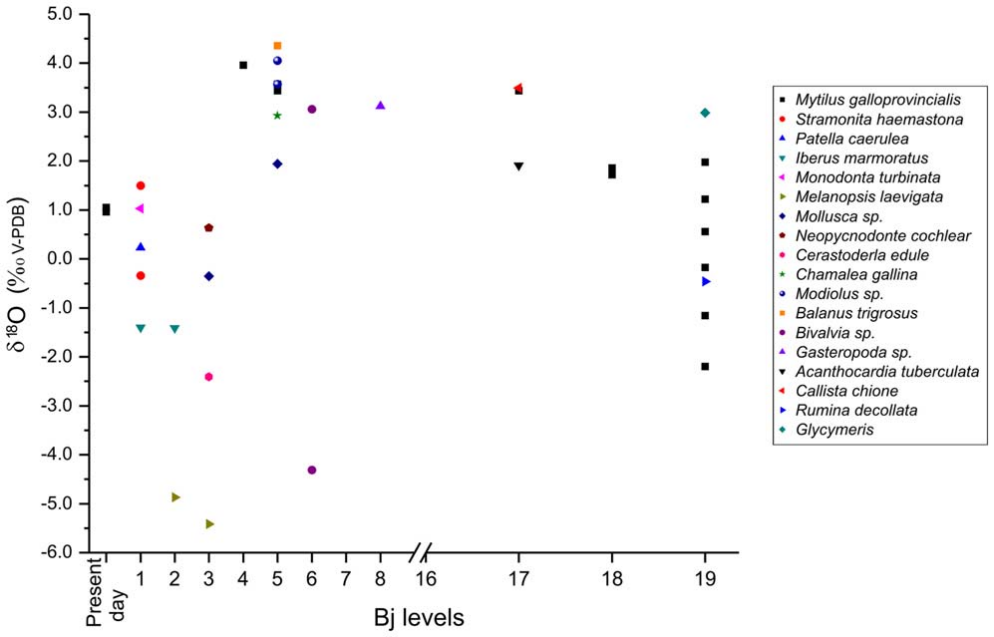
Stable oxygen isotopic composition of shells from Bajondillo Cave and Malaga coast. Bajondillo Cave: aragonitic and calcitic mollusk shells and two present-day specimens of *Mytilus cf. galloprovencialis* from the coast of Malaga.

**Figure 7:**
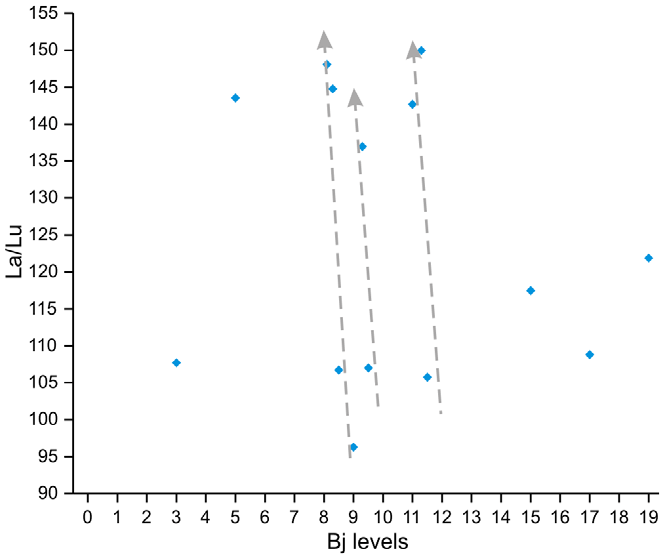
Bulk sediment La/Lu ratios from Bajondillo Cave. Grey arrows reveal the increase of La/Lu values across eolian sand-rich layers from Bajondillo Cave.

In order to test the consistency and coherence, both chronological and contextual, of the Bj_19_ archaeological deposit, a series of isotopic studies were carried out (see Materials and Methods for details):

- δ^13^C values: these ranged between −29‰ to −23‰, typical for C3 plants in soils [13]. The most negative values roughly corresponded to the Holocene and the MIS 5 and MIS 6 stages whereas enrichments in the heavy carbon isotope fraction corresponded to MIS 2, MIS 3 and MIS 4 (Figure 2). Sharp variations in δ^13^C values during the various marine isotopic stages link with abrupt climatic events that in addition to Heinrich events, include the Dansgaard-Oeschger cold stadial and the relatively warm interstadial cycles during the glacial isotopic periods. The δ^13^C trend plotted to the age (Figure 2) showed a clear correlation with our palynological data and corroborated the climatic influence on the δ^13^C signal. Although the temporal resolution of cave deposits is generally considered to be coarse when compared with other paleoclimatic records, the correlations between the δ^13^C values and the sedimentary facies at Bajondillo Cave are also coherent, evidencing positive values in rich gelifracted levels generated during cold and arid episodes, and negative values during moments of active travertine formation. The postulated humid conditions during the Holocene and MIS 5 inferred from the δ^13^C values are also coherent with independent paleotemperature and paleohumidity records in this region such as the deep-sea cores on the Sea of Alboran [14] and speleothems formed during the MIS 6 to MIS 2 stages [15].
- As has been evidenced in other regions, the amplitudes of the isotopic variations in our recent specimens of *M. galloprovincialis* correspond to the annual amplitude of the surface seawater temperature in the area [16]. In the case of the archaeological specimens (Figure 6), values clearly reflected cold and salty seawater conditions, in the case of the glacial MIS 4, MIS 3 and MIS 2 stages, and the warmer conditions that are associated with the terminal MIS 6, and the MIS 5 and MIS 1 stages. The recorded *M. galloprovincialis* values of *ca.* 3.5‰ from Bj_17_ (MIS 4) and Bj_5_ (Early Holocene) are comparable to Late Holocene values of *M. galloprovincialis* from the North Atlantic (i.e., *ca.* 3.0‰) [17]. The major shift in the isotopic values recorded for Bj_4-3_ would correspond with an approximate age of 7.4 cal ka BP. Such intra-Holocene variation is coherent with the last of the major oceanographic reconfigurations that occurred in the western Mediterranean at that time [18]–[19], and conforms with the prevalence of cold faunas in the region during the early Holocene [20]–[21]. The variability also indicates that isotopic variation within this coastal fauna reflects local water mass shifts, rather than global isotopic seawater changes [19]. For these reasons, the isotopic data are in agreement with both the dates of the levels where the samples were taken and with previously studied regional paleo-environmental proxies. Most important is the fact that the oxygen isotopic data provided by the shells sampled from Bajondillo Cave (Bj_19-17_) evidence that these were contemporaneous with the deposits in which they were retrieved.
- Microstratigraphic studies at Bajondillo Cave point to a progressive increase in the eolian input in levels Bj_11_, Bj_9-8_ [6] that contrasts with levels Bj_3_, Bj_5_, Bj_15_, Bj_17_ and Bj_19_, where no eolian sand-rich layers have been detected. For such reason, a detailed sampling was undertaken in all these levels to geochemically typify the nature of the eolian input. Our results evidence an increase in the La/Lu ratio for the sand-rich levels and low La/Lu values at Bj_17_ and Bj_19_, where eolian inputs were not described by the microstratigraphic study (Figure 7).

**Figure 8:**
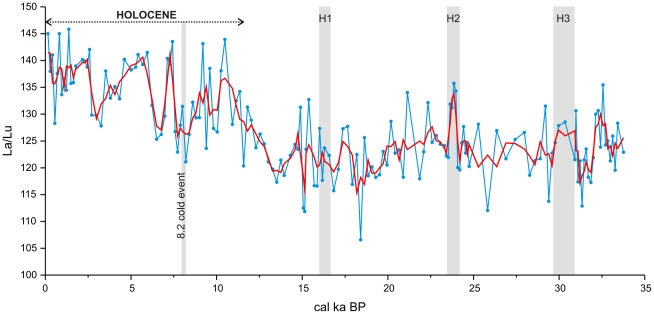
La/Lu ratio versus age, obtained in bulk sediments from site 300G (Alboran Sea basin). The red line depicts the 3-point smoothing average. Light grey vertical bars signal cold periods, including Heinrich events (H1–H3) and the 8.2 cal ka BP cold episode.

Similar La/Lu variations can be observed in the nearby marine sediments studied in previous analyses. In this way, during glacial periods, the La/Lu ratio featured low values in the marine sediments from site 300G on the Alboran Sea (Figure 8). Given that an increase in La/Lu values develops during Heinrich events that represent cold and arid episodes with intense atmospheric circulation [22], the high La/Lu ratios can be probably linked with changes taking place in the Saharan eolian source. Indeed, radiogenic isotope studies on marine cores from the western Mediterranean Sea identify the Southern Sahara/Sahelian region as the main eolian source during MIS 2, MIS 3, MIS 4 and MIS 6, and the Northern Sahara during MIS 1 and MIS 5 [23]. In addition, the high La/Lu values obtained for the eolian deposits inside the cave appear to take place almost directly and concurrently with low levels of anthropic artifacts.

In conclusion, and despite the limitations of cave deposits as paleoclimatological records, the high La/Lu ratios are associated with eolian inputs deposited during glacial periods (i.e., MIS 2 and MIS 3) when a less intense occupation is documented at Bajondillo Cave and glacial and arid conditions prevailed.

### Pollen record

The palinological record (Figure 5) shows that Bj_19_ is dominated by Mediterranean taxa, mainly evergreen *Quercus*, pines, and elements from the xerothermophilous *maquis*. The presence of mesophilous trees and of hydrophilous taxa suggests a cold to temperate and dry to sub-humid climate. Pollen spectra from Bj_18_ exhibit a similar pattern, with a slight reduction of the steppe taxa and an appearance of significant thermophilous taxa such as *Cosentinia*, *Withania* and *Selaginella denticulate*. This suggests temperate to thermic and sub-humid conditions. The glacial period that corresponds to Bj_17_ is characterized by a marked increase of the steppe taxa and of the Poaceae, a marked decrease in the arboreal cover of evergreen oaks, and the disappearance of humid (i.e., mesophilous trees and hydro-hygrophilus taxa) and thermic (*Cosentinia*, *Withania* and *Selaginella denticulata*) elements, indicating the existence of very cold and arid conditions.

### Synthesis and implications

It has been recently claimed that the expansion of the diet to incorporate foods of marine origin constitutes a key adaptation of modern humans (*Homo sapiens*) and a reflection of a major shift taking place in the cognitive capabilities of the species [24]–[29]. In addition, this coastal adaptation, exemplified by marine mollusk exploitation, has been postulated as crucial to a potential coastal route of modern humans out of Africa to Asia via the Red Sea after ∼120 ka [30]–[31]. However, the earliest evidence reveals that coastal resource exploitation was already present 164 ka in Pinnacle Point, South Africa [25], [29], [32], long before these migrations took place, and the claims that intensive shellfish collecting is a trait of modern humans disregard those studies documenting Neanderthal use of shellfish during MIS 3 [1].

The chronological data from Bj_19_ presented in this paper confirm the existence of shellfish gathering at Bajondillo Cave as early as *ca.* 150 ka BP (MIS 6). Such a statement derives from the coherence of the archaeological sequence substantiated on 29 datings through radiocarbon, TL and U series methods. Different proxies (e.g., pollen, isotopic content, detrital elements and lithostratigraphic features) have been studied at Bajondillo Cave allowing to reconstruct paleoenviromental conditions during cave occupation. In addition, δ^18^O values of the bivalve shells and δ^13^C values from organic matter (i.e., C3 plant remains) in the soil, together with inorganic proxies (La/Lu ratio) measured in the detrital fraction from levels Bj_19-17_, appear fully consistent with environmental conditions associated with MIS 6-MIS 4 stages in the region (Figure 2).

The data presented provide compelling evidence that shellfish harvesting was part of the Neanderthal trophic niche at essentially the same time when Modern Humans were also exploiting the coast in South Africa [25]–[29], [32]. Although such behavioural convergence may strike some as remarkable it is probably far from extraordinary, given the use of stones for breaking open oysters, crabs and other coastal fauna by certain primates in Asia, first documented by Carpenter in 1887 and “rediscovered” in the aftermath of the 2004 tsunami [33]–[34].

From such a perspective, and although undisputable evidence is presently lacking, it might well be that early hominids started exploiting marine mollusks along the Mediterranean shores as early as the Middle Pleistocene. Reflecting on it, as well as on data such as the 800 ka-old stone tools from the island of Flores [35]–[36], the contraction of Neanderthal populations [37] after this first documentation of their adaptation to the coast runs counter to some of the expectations of the proposed model of territorial expansion for modern humans out of Africa.

The coincidence of dates at Bajondillo Cave with those from Pinnacle Point in South Africa, suggest that shellfish gathering reveals yet another case where Neanderthals and Modern Humans might have been following parallel behavioural trajectories, with different evolutionary outcomes. For that reason, and also because shellfish gathering appears to be totally disconnected from the symbolic sphere, those data reinforce our suspicion that the coastal adaptation, however important it might have been at the local level of specific populations, may be yet another overrated phenomenon in the list of behaviors long considered to represent modernity [24], [30]–[31], [38].

## Materials and Methods

### Archaeological Methods

Sediment volumes were measured during excavation, and bulk samples of sediment were taken from every unique stratigraphic unit. All observed finds were located in three dimensions, whereas the rest were sequentially captured by nested 10-mm→5-mm→2-mm and 1-mm→0.5-mm→0.1-mm dry-sieving. Screened materials were packed in plastic bags and transported to the laboratory. Remains of archaeological materials each have their specific labelling system. Finds were sorted in the laboratory and provided to the appropriate specialist for analysis (lithics, mammals, shellfish, etc). Lithics were analysed by a combination of typological, technological, raw material, use wear analysis and metrical variables from a database. Shellfish and mammals were identified by comparison to known modern specimens. No portion of any artifact image was retouched or otherwise edited. Archaeological and stratigraphic interpretations were derived from a combination of field-based, macro-stratigraphic observations, computer analysis of mapped stratigraphic units, analyses of plotted find distributions, and micromorphology.

The paleoclimatic interpretation is based on a combination of geomorphological, sedimentary micromorphology, pollen, geochemical, isotopic, stratigraphic and chronological data. Noteworthy is the fact that the archaeological materials presented as well as 28 of the 29 available datings (Table 2), the 22 sedimentary micromorphology samples, 114 pollen samples, and the 156 samples selected for geochemical and isotopic analyses (Figure 2), all derive from the same profile and from a restricted sector of Bajondillo Cave, allowing for a very fine correlation of the various data sets.

### Pollen analysis methods

A total of 114 samples were analyzed. The pollen diagram (Figures 2, 5) comprises, for each sample, a minimum of 200 pollen grains and 20 recorded taxa to enable statistically reliable inferences [39]–[40]. The relative values for each taxon, whether arboreal, shrub or herbaceous, were obtained from the sum of the absolute values for each taxon referred to a pollen total. This total does not include hygrophilous taxa, cryptogam spores, undetermined pollen grains or Cichorioidea because of their hypothetical overrepresentation in sedimentary deposits due to their zoophyllic character [41].

### Organic matter C isotopic analyses

A total of 96 samples were taken at approximately 5 to 10 cm intervals along the entire archeological sequence. Carbon isotope ratios of bulk organic matter were measured at the Instituto Andaluz de Ciencias de la Tierra (CSIC-UGR). After carbonate removal on a 1∶1 HCl solution, δ^13^C values of organic matter were measured in selected samples by means of an EA-IRMS elemental analyzer connected to a Finnigan MAT 251 mass spectrometer. Results are expressed in *δ* notation (‰), using the international Vienna Pee Dee Belemnite standard (V-PDB). The standard deviations are of 0.1‰ for δ^13^C in organic matter.

The carbon isotopic composition of organic matter reflects the dynamics of carbon assimilation during photosynthesis and the isotopic composition of the carbon source, which depends on environmental conditions [42]. The δ^13^C values of organic matter in lake sediments are frequently used to distinguish among the different organic matter sources, in particular between terrestrial and aquatic plants. They can further differentiate between different types of land plants (C3 and C4) and be used to measure algal productivity [43]. In cave sediments, where mainly C3 plants are present, δ^13^C values can be correlated with humidity. Water stressed ecosystems are enriched in ^13^C, reaching up to −22‰ when compared with the average C3 value of *ca*. −27‰ [12], and this allows for the use of δ^13^C as a paleohumidity proxy [44].

### Carbonate shell isotopic analyses

A total of 32 shells from Bajondillo Cave and two present-day mussels (*Mytilus galloprovincialis*) from the Malaga coast collected in 2004 at the Maro-Cerro Gordo Nature Park were analyzed in order to reveal their oxygen isotopic composition (Table 6). Of the former, 12 samples corresponded to mussels from Bj_19-17_ and Bj_5-4_ (Table 3 and Figure 6), the remaining samples incorporating species of both gastropods and bivalves (Table 6). Only adult specimens have been sampled in order to avoid the isotopic fractionations of kinetic and metabolic origin that affect parts of the shells in the juveniles of many species [45]. XRD analysis revealed the original aragonitic/calcitic composition in all cases evidencing that none of these shells had been altered diagenetically. Four samples were micro-drilled for high resolution examination.

**Table 6:** Stable oxygen isotopic composition of shells from Bajondillo Cave and Malaga coast.

Sample	*M.g.*	*S.h.*	*P.c.*	*Ib.m.*	*M.t.*	*M.l.*	Moll.	*N.c.*	*C.e.*	*Ch.g.*	*Mod.*	*B.t.*	*B.*	G.	*A.t.*	*C.ch.*	*R.d.*	*Gi.*
P.D.	0.96	-	-	-	-	-	-	-	-	-	-	-	-	-	-	-	-	-
	1.05	-	-	-	-	-	-	-	-	-	-	-	-	-	-	-	-	-
Bj_1_	-	−0.34	-	-	-	-	-	-	-	-	-	-	-	-	-	-	-	-
	-	-	0.23	-	-	-	-	-	-	-	-	-	-	-	-	-	-	-
	-	-	-	−1.40	-	-	-	-	-	-	-	-	-	-	-	-	-	-
	-	1.5	-	-	-	-	-	-	-	-	-	-	-	-	-	-	-	-
	-	-	-	-	1.03	-	-	-	-	-	-	-	-	-	-	-	-	-
Bj_2_	-	-	-	−1.41	-	−4.87	-	-	-	-	-	-	-	-	-	-	-	-
Bj_3_	-	-	-	-	-	-	−0.35	-	-	-	-	-	-	-	-	-	-	-
	-	-	-	-	-	-	-	0.63	-	-	-	-	-	-	-	-	-	-
	-	-	-	-	-	-	-	-	−2.41	-	-	-	-	-	-	-	-	-
	-	-	-	-	-	−5.42	-	-	-	-	-	-	-	-	-	-	-	-
Bj_4_	3.96	-	-	-	-	-	-	-	-	-	-	-	-	-	-	-	-	-
Bj_5_	-	-	-	-	-	-	-	-	-	2.93	-	-	-	-	-	-	-	-
	-	-	-	-	-	-	-	-	-	-	4.05	-	-	-	-	-	-	-
	-	-	-	-	-	-	1.94	-	-	-	-	-	-	-	-	-	-	-
	-	-	-	-	-	-	-	-	-	-	-	4.36	-	-	-	-	-	-
	3.57	-	-	-	-	-	-	-	-	-	3.57	-	-	-	-	-	-	-
	3.44	-	-	-	-	-	-	-	-	-	-	-	-	-	-	-	-	-
Bj_6_	-	-	-	-	-	-	-	-	-	-	-	-	−4.31	-	-	-	-	-
	-	-	-	-	-	-	-	-	-	-	-	-	3.06	-	-	-	-	-
Bj_8_	-	-	-	-	-	-	-	-	-	-	-	-	-	3.12	-	-	-	-
Bj_17_	-	-	-	-	-	-	-	-	-	-	-	-	-	-	1.91	-	-	-
	-	-	-	-	-	-	-	-	-	-	-	-	-	-	-	3.49	-	-
	3.44	-	-	-	-	-	-	-	-	-	-	-	-	-	-	-	-	-
Bj_18_	1.72	-	-	-	-	-	-	-	-	-	-	-	-	-	-	-	-	-
	1.86	-	-	-	-	-	-	-	-	-	-	-	-	-	-	-	-	-
Bj_19_	−1.16	-	-	-	-	-	-	-	-	-	-	-	-	-	-	-	-	-
	1.98	-	-	-	-	-	-	-	-	-	-	-	-	-	-	-	-	-
	−2.20	-	-	-	-	-	-	-	-	-	-	-	-	-	-	-	-	-
	1.22	-	-	-	-	-	-	-	-	-	-	-	-	-	-	-	-	-
	−017	-	-	-	-	-	-	-	-	-	-	-	-	-	-	-	-	-
	0.56	-	-	-	-	-	-	-	-	-	-	-	-	-	-	-	-	-
	-	-	-	-	-	-	-	-	-	-	-	-	-	-	-	-	−0. 46	-
	-	-	-	-	-	-	-	-	-	-	-	-	-	-	-	-	-	2.99

Codes as follows: P.D.: Two present-day (year 2004) specimens of *Mytilus galloprovincialis* from the coast of Malaga. Species M.g. (*Mytilus galloprovincilis*), S.h. (*Stramonita haemastoma*), P.c. (*Patella caerulea*), Ib.m. (*Iberus marmoratus*), M.t. (*Monodonta turbinata*), M.l. (*Melanopsis laevigata*), Moll. (Mollusk sp), N.c. (*Neopycnodon cochlear*), C.e. (*Cerastoderma edule*), Ch.g. (*Chamalea gallina*), Mod. (*Modiolus* sp), B.t. (*Balanus trigosus*), B (Bivalvia sp), G (Gastropoda sp), A.t. (*Acanthocardia tuberculata*), C.ch. (*Callista chione*), R.d. (*Rumina decollata*), Gi. (*Glycymeris* sp).

Shells were dried at 50°C and, after a mechanical removal of their most superficial layers, were ground to a fine powder. Carbon dioxide was extracted from the calcite using 100% phosphoric acid for 5 h in a thermostatic bath at 50°C [46]–[47]. A Pyrex microline was used for gas purification. The carbon and oxygen stable isotopes analyses were conducted in a Finnigan MAT 251 mass spectrometer from the Instituto Andaluz de Ciencias de la Tierra (CSIC-UGR, Granada). Isotopic results are reported in the standard delta (δ) notation in parts per thousand (‰) relative to the international V-PDB standard [48]. All samples were compared to a reference carbon dioxide obtained from a calcite standard (internal and international standard) prepared at the time that samples were taken for analysis. The experimental *δ* ^18^O error for calcite was less than ±0.1‰. Carrara and EEZ-1, previously compared with the international standards NBS-18 and NBS-19, were used as the internal standards. For the micro-drilled samples, the ca. 30 µg of powder collected for each sample were analyzed using a mass spectrometer (IsoPrime; GV Instruments Ltd.) with a Multiprep individual acid bath carbonate precipitation device, at the Japan Agency for Marine-Earth Science and Technology (JAMSTEC). The reproducibility of standard materials run higher than 0.08‰ for δ^18^O.

The micro-drilled present-day *M. galloprovincialis* samples exhibited a range of δ^18^O values between 0.428‰ and 1.344‰, with averages ranging between 0.96‰–1.05‰ (Figure 2, Table 6). In the archaeological mussels, δ^18^O values ranged between −2.2‰ and 4.0‰. The lowest values (<2‰) corresponded to Bj_19-18_ and present day samples and the highest values (>2‰) corresponded to Bj_17_, Bj_15_ and Bj_5-4_ (Figure 6). When all samples were compared, we obtained a similar distribution with low values for Bj_19-18_, Bj_3- 1_, and present-day samples, and high values for the samples from the remaining archeological levels (Table 6). Variability in δ^18^O values for other species recovered from the same archeological level was very low as recorded for *Callista chione* and *Acanthocardia tuberculata* in level Bj_17_ and for *M. galloprovincialis* in levels Bj_18_ and Bj_5_ (Figure 6). Only in level Bj_19_ did we obtain a wide range in δ^18^O values for *M. galloprovincialis* (i.e., from −2.2‰ to 1.9‰).

### Bulk sample geochemical analysis

In order to determine the source area of the eolian deposits, the rare earth element La/Lu ratio has been used as a proxy in both the Mediterranean area [49]–[50] and the archaeological record [51]. In the region of study, the La/Lu ratio has been specifically used to discriminate eolian inputs of the African craton from those of the European margin [52].

Previous sedimentological analyses evidenced detritus material associated with calcareous tufa, eolian deposits and objects of anthropic origin to constitute the main sedimentary infilling at Bajondillo Cave [6]. A total of fourteen samples were performed using inductively coupled plasma-mass spectrometry (ICP-MS) previous to a HNO_3_+HF digestion. Measurements were taken in triplicates by spectrometry (Perkin-Elmer Sciex Elan 5000) using Re and Rh as the internal standards. The instrumental error is of ±2% and ±5% for elemental concentrations of 50 ppm and 5 ppm, respectively [53].

### U/Th bone datings

Due to the very low uranium content of living bones and because fossil ones acquire uranium post-depositionally, bones would not, in principle, be considered an ideal material for U/Th dating. Still, and although their uptake mechanism is not well understood, their high affinity for uranium, one or two orders of magnitude higher than that of authigenic carbonates, has evidenced that they can nevertheless be useful for U/Th dating purposes [54]–[55]. In an ideal scenario, bone would acquire uranium after burial in an early uptake (EU) and then would remain as a closed system. This is often assumed to be the case for the younger bone samples (ca. <20 ka). Under such assumption, their ^14^C dates should be concordant with their U/Th age [56]–[58]. Older samples, on the other hand, often provide U/Th ages that are too young, indicating a later U assimilation with time [56], [59]. In order to incorporate bones for dating purposes it is thus critical to determine first their U-uptake mechanism. Bischoff *et al.* derived two mathematical models of linear uptake for U–series dates on bone samples [60]. The first model assumes a linear uptake of ^238^U and ^234^U followed by decay to ^230^Th with time. At t = 0 (i.e., initial conditions), the bone would be free of ^238^U, ^234^U and ^230^Th. The second model assumes that the bone sample fixes a finite amount of ^238^U and ^234^U soon after burial, and thereafter continues to absorb ^234^U greatly in excess of ^238^U. According to this second model, at t = 0, the bone has ^238^U and ^234^U in a given ratio but no ^230^Th. This model applies to situations where fossil bones have ^234^U/^238^U activity ratios considerably in excess of the value in the surrounding waters [61].

Bischoff et al.'s mathematical equations [60] have been used to develop a computer program in order to evaluate the age of three bone samples assuming three U-uptake situations: (a) U/Th nominal ages (EU) [60], (b) a linear uptake of U (LU84), and (c) an early uptake of U followed by a linear uptake of ^234^U (LU4). This program also incorporates the error implicit in the mathematical equations.

Three bones were analyzed for U and Th concentrations. One gram of each bone was dissolved in nitric acid. The solution was afterwards separated from solid residue by filtration with a filter of 0.45 µm pore size. This solution was used for both U and Th analysis. An iron carrier (FeCl_3_), and a well known amount of ^232^U and ^229^Th for yield calculations, were added to the solution. Precipitation of the iron hydroxides for pH values above 9 was carried out using concentrated ammonia. Uranium was separated from Th and P with a solvent extraction method that used tributylphosphate (TBP) and xilene as the organic phase. The uranium fraction was then ready for electroplating.

The thorium fraction needed to be purified. After precipitation of the iron hydroxides for pH values above 9, the precipitate was dissolved in HCl. Thorium was separated from P through precipitation at a pH = 3.5. After Th precipitated a minimal fraction P remained in solution. For such reason, the process was carried out two additional times in order to insure the elimination of all of the P. To further purify Th, an anion exchange resin (i.e., Dowex AG1-X8) was used. After completion of this procedure the solution was ready for electroplating.

The electroplating of uranium and thorium was performed for one hour at 1.2 A onto stainless steel discs. One minute before switching off the current, 1 ml of NH_3_ was added. The discs were measured with alpha spectrometry. The alpha spectrometer was equipped with PIPS detectors.

This method has provided good U and Th chemical recoveries, in both cases above 50% (Tables 2 and 5).
